# A Biomechanical Re-Examination of Physical Activity Measurement with Accelerometers

**DOI:** 10.3390/s18103399

**Published:** 2018-10-11

**Authors:** Jonatan Fridolfsson, Mats Börjesson, Daniel Arvidsson

**Affiliations:** 1 Center for Health and Performance, Department of Food and Nutrition, and Sport Science, University of Gothenburg, 40530 Gothenburg, Sweden; mats.brjesson@telia.com (M.B.); daniel.arvidsson@gu.se (D.A.); 2 Institute of Neuroscience and Physiology, University of Gothenburg, 40530 Gothenburg, Sweden; 3 Sahlgrenska University Hospital/Östra, 40530 Gothenburg, Sweden

**Keywords:** ActiGraph, axivity, filtering, children, adults

## Abstract

ActiGraph is the most common accelerometer in physical activity research, but it has measurement errors due to restrictive frequency filtering. This study investigated biomechanically how different frequency filtering of accelerometer data affects assessment of activity intensity and age-group differences when measuring physical activity. Data from accelerometer at the hip and motion capture system was recorded during treadmill walking and running from 30 subjects in three different age groups: 10, 15, and >20 years old. Acceleration data was processed to ActiGraph counts with original band-pass filter at 1.66 Hz, to counts with wider filter at either 4 or 10 Hz, and to unfiltered acceleration according to “Euclidian norm minus one” (ENMO). Internal and external power, step frequency, and vertical displacement of center of mass (VD) were estimated from the motion capture data. Widening the frequency filter improved the relationship between higher locomotion speed and counts. It also removed age-group differences and decreased within-group variation. While ActiGraph counts were almost exclusively explained by VD, the counts from the 10 Hz filter were explained by VD and step frequency to an equal degree. In conclusion, a wider frequency filter improves assessment of physical activity intensity by more accurately capturing individual gait patterns.

## 1. Introduction

Physical activity is related to health, and investigating this relationship requires accurate measurement methods. The use of accelerometers has increased extensively during the last decade to provide objective measures of physical activity. Several methods for processing raw acceleration data have been developed. Among them, ActiGraph counts is by far the most common, being used in >50% of published studies [1,2]. The details of this method was earlier inaccessible information, but Brønd et al. have recently managed to replicate the process using an alternative monitor, providing insights into these details [3]. ActiGraph counts are an aggregation of the absolute acceleration measured from an ActiGraph accelerometer typically placed at the hip. To enable further interpretation, counts are calibrated to estimate the time spent performing physical activity with different intensities [1]. Previous research has demonstrated that ActiGraph counts peak at about 10 km·h^−1^, although the acceleration continues to increase with higher speeds [4]. Further, children produce fewer counts than adults when walking and running at the same speed [5,6]. In contrast, higher values have been observed in children than in adults with the unfiltered raw acceleration metric Euclidian Norm Minus One (ENMO) [7]. These incongruent results warrant re-examination of accurate acceleration processing into activity intensity metrics.

ActiGraph data processing includes a band-pass frequency filter with a low-pass half-power cut-off frequency at 1.66 Hz, attenuating the parts of the signal with higher frequency [3]. When investigating the frequency spectrum of the acceleration signal during locomotion, the most relevant information ranges up to 4 Hz, corresponding to step frequency, but there seems to be locomotion-related information up to 10 Hz [8]. The locomotion-related power also seems to be distributed within these ranges [9]. Comparing accelerometer data to simultaneous measurements with a 3D motion capture system or force plates indicates that an optimal filter for accelerometer data might be around 8–10 Hz [10,11]. Children move with higher step frequency than adults at a given speed [12,13]. The step frequency during running among adults is around 2.5 Hz, whereas children have a step frequency around 3 Hz at the same speed [13]. Consequently, there is a differential measurement error with ActiGraph counts depending on locomotion intensity (running vs. walking) and age group (children vs. adults) caused by the band-pass frequency filter.

From a biomechanical perspective, the total mass-specific work continuously increases with locomotion speed and is the same across age groups [12,14]. The mass-specific total work can be divided into external, i.e., moving the body’s center of mass in relation to the environment, and internal, i.e., moving the limbs in relation to the body’s center of mass. The internal work is proportional to the step frequency, whereas the vertical component of the external work is proportional to the vertical displacement of the center of mass but inversely proportional to the step frequency. Adults produce more external work compared to children because of longer strides, but children compensate this with a higher step frequency and therefore more internal work [12,14]. Because of the relation between step frequency and work components, a narrow frequency filtering of accelerometry data would theoretically filter out more of the acceleration related to internal work compared to external. Widening the frequency filter enough to capture the whole range of normal step frequencies and other signals relevant to human locomotion could possibly more accurately represent the total work performed across activity intensities from a biomechanical perspective.

Therefore, the aim of this study was to explore the effect of frequency filtering level when processing raw accelerometer data from children and adults moving at the same speed and relate it to biomechanical variables. Our hypothesis was that widening the frequency filter will change the relationship between activity intensity and accelerometer output to become more similar to what is expected from the biomechanical literature and generate more similar accelerometer output by age.

## 2. Materials and Methods

### 2.1. Study Design

The calibration study design was a controlled lab setting with subjects from three different age groups walking and running on a treadmill. Acceleration data were recorded using body-mounted sensors and compared to biomechanical variables calculated from a motion capture system.

### 2.2. Study Sample

The study sample consisted of 30 subjects equally distributed between each of the following age groups: children (age 10), adolescent (age 15), and adults (age > 20). Subjects were members of local sports clubs, institutional staff and students, and personal contacts, and they were recruited through oral information and by written announcements through email, aiming for an even gender distribution. Subjects received prior information about the testing procedure, and the children were also shown pictures to illustrate the process. Written informed consent was obtained from the subjects in the adult group, from the subjects and the subjects’ caregivers in the adolescent group and from the subjects’ caregivers in the children group. The study was approved by the regional ethics committee in Gothenburg (No. 1026-17).

### 2.3. Study Protocol

**Figure 1:**
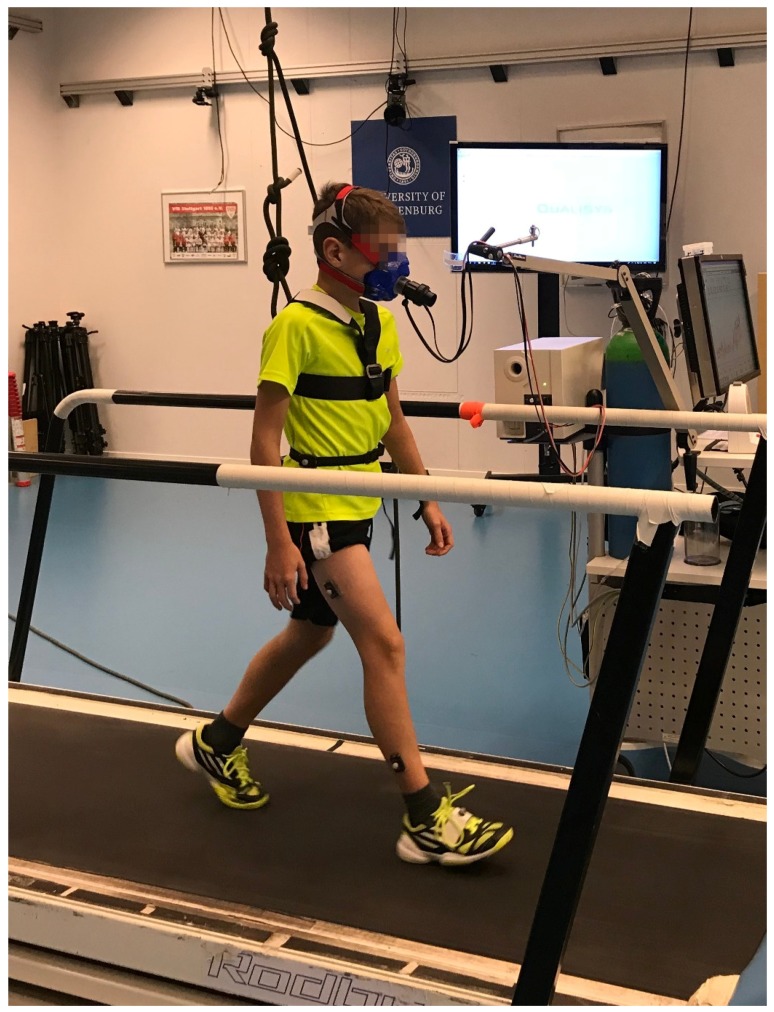
Example of the experimental setup.

Subjects were instructed to not consume any food for 3–4 h immediately prior to the experiment and to avoid strenuous physical activities on that day [15]. Anthropometrics were measured consisting of body height and weight as well as length of the left upper arm and forearm and the right thigh and shank. Reflective markers were attached with adhesive tape lateral to the center of mass of the extremity segments measured [16]. Subjects also wore an elastic band around the waist with an accelerometer taped to it at the right hip and reflective markers attached to the accelerometer and to the elastic band at the center of the lower back. Children in the youngest age group wore a safety harness tied to a rope in the ceiling as a safety measure. Figure 1 shows an example of the experimental setup. To synchronize the accelerometer with the timing of the test protocol, five vertical jumps were performed. Thereafter, the treadmill protocol with four minutes at each speed started with walking at 3, 4, 5, and 6 km·h^−1^, followed by running at 8, 10, and 12 km·h^−1^. The protocol was performed continuously, with the youngest age group expected to manage running at 10 km·h^−1^, while the older two ran at 12 km·h^−1^. One subject in the youngest group did not manage running at 10 km·h^−1^. Therefore, data from only one running speed was used in the analyses for this subject. All data processing was performed in Matlab R2017b (MathWorks, Natick, MA, USA).

### 2.4. Collection and Processing of Acceleration Data

The accelerometer used was the AX3 (Axivity Ltd., Newcastle upon Tyne, UK), a small (23 × 32.5 × 7.6 mm, 11 g), waterproof, open-source, and relatively cheap triaxial accelerometer. The OmGUI software (Axivity Ltd., Newcastle upon Tyne, UK) was used for sensor initialization and data extraction. Sampling frequency and sensitivity was set to 100 Hz and ± 8 g, respectively. The autocalibration method described by van Hees et al. [17] was used to calibrate each axis, and the data was resampled to 30 and 60 Hz.

Acceleration resampled to 30 Hz was processed to original ActiGraph counts (AG) with a low-pass cut-off at 1.66 Hz and activity counts with modified low-pass cut-offs at 4 and 10 Hz, respectively (AC4, AC10). The processing of raw acceleration to AG counts described by Brønd et al. consists of the following steps performed on the data from each axis [3]:

1. Aliasing filter 0.01–7 Hz
2. ActiGraph filter 0.29–1.66 Hz
3. Resampling to 10 Hz
4. Truncation at 2.13 g
5. Rectification
6. Dead-band at 0.068 g
7. Conversion to 8-bit resolution
8. Aggregation, initially to 1-s epochs.

With the modified counts, no aliasing filter, additional resampling, or truncation was performed; instead, only the following steps were performed as previously described [8]:

1. Band-pass filter with wider bandwidth
2. Rectification
3. Dead-band at 0.068 g
4. Conversion to counts, 2.13 g to 8-bit resolution
5. Aggregation, initially to 1-s epochs.

The wider filters were implemented as digital fourth order Butterworth band-pass filters with the same half-power high-pass cut-off as the standard ActiGraph filter but with altered half-power low-pass cut-offs at 4 and 10 Hz. The original AG method converts the data in the range of 0–2.13 g to whole numbers between 0 and 127 because of the 8-bit resolution, which is equal to dividing the acceleration by 0.0166. To facilitate comparability with AG counts, the acceleration data from the modified methods were also divided by 0.0166. The data from the count-based methods were initially aggregated to 1-s epochs, followed by calculation of the vector magnitude of the counts from each axis according to the Euclidean norm. To enable investigation of the results from Hildebrand et al. [7], the 60 Hz data was similarly processed using the unfiltered acceleration metric ENMO [18]. ENMO was included to represent a method without band-pass filtering. The acceleration captured between the times 2:45 and 3:45 was used in order to ensure that the subject had time to familiarize themselves with the speed on the treadmill to walk and run in their habitual manner. The ENMO acceleration was averaged for this time, and the counts were summed up to represent a 1-min epoch.

### 2.5. Biomechanical Variables

Position data of the reflective markers was retrieved from the Qualisys motion capture system (Qualisys AB, Gothenburg, Sweden) to calculate step frequency, vertical displacement of the estimated center of mass (VD), and internal and external power and to create a frequency spectrum of the acceleration as a reference to the accelerometer data. The position of the makers was tracked by 17 high-speed cameras at a sampling rate of 400 Hz using infrared exposure. The mean of all biomechanical variables was calculated for the data between the times 2:45 and 3:45 at each locomotion speed. The system was calibrated before each session according to the producer’s instruction. The mean step frequency and VD was calculated from the marker at the lower back. The position data of the lower back marker was filtered using a fourth-order Butterworth low-pass filter with a half-power frequency at 5 Hz after which the local maximum and minimum points were identified, and the vertical distance and time of each gait cycle were averaged. As a reference to the acceleration signal captured by the accelerometer at the hip, a power frequency spectrum of the acceleration of the marker attached to the accelerometer was calculated. First, a fourth-order Butterworth low-pass filter with a half-power frequency at 20 Hz was used, followed by differentiating the data with respect to time twice to acceleration and calculating the power frequency spectrum using the Matlab pwelch function.

An approximation of the internal and external mass-specific power (kilojoule per kilogram per second) was also calculated from the motion capture data using a method that was modified from Cavagna [19]. The center of mass of the body and each extremity segment was approximated by the marker on the lower back and the markers on the extremities, respectively. Because only one marker on each segment was used, the rotational inertia could not be accounted for, and the marker on the lower back was considered the center of mass instead of the true one. A fourth-order Butterworth low-pass filter with a half-power frequency at 10 Hz was used to filter the trajectories of the marker positions. The instantaneous mass-specific external power (P·m^−1^) was calculated by multiplying the vector magnitude of the velocity and acceleration of the marker on the lower back according to the following theorem derived from classical mechanics:

P·m^−1^ = v·a, (1)

where the velocity and acceleration were retrieved by differentiating the position with respect to time. The instantaneous mass-specific internal power was calculated from the relative weight, speed, and acceleration of the segments and multiplied by two because only one side was measured. The relative segment weight in relation to body weight varies with age, which was accounted for [14]. The relative velocity and acceleration of the upper arm, forearm, thigh, and shank was retrieved from the relative position of these markers from the marker at the lower back.

### 2.6. Statistics

The results were visually presented by plotting separate linear regressions of the accelerometer output and power with regard to speed for walking and running. Two separate linear regressions were used because of the fundamental differences between the mechanics of running and walking [20]. The coefficient of variation (CV) of the accelerometer output was calculated for each treadmill speed, age group, and method. Separate one-way ANOVAs for each speed and processing method were performed with the accelerometer output as an independent variable and age group as a dependent variable. A total of 28 ANOVAs were performed. To further investigate the effect of biomechanical factors on the acceleration output, a multiple linear regression was performed between the output from the different data processing methods as dependent variables and VD and step frequency as independent variables, treating each speed from each person as a separate observation.

## 3. Results

**Figure 2:**
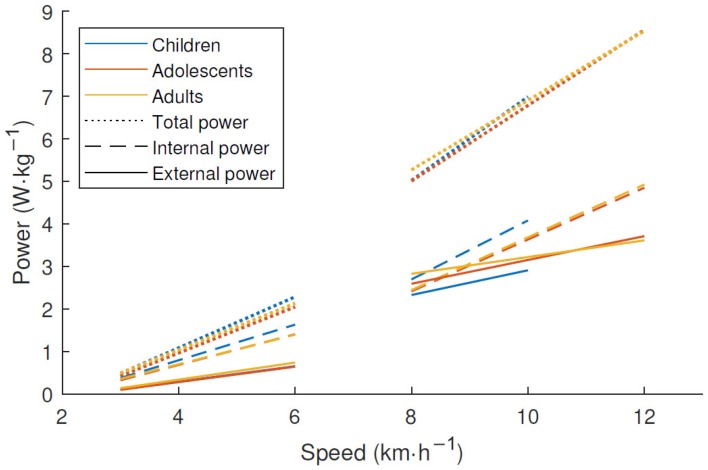
Linear regressions of total, external, and internal mass-specific power estimated from motion capture data during walking and running separately with 10 subjects in each age group.

**Figure 3:**
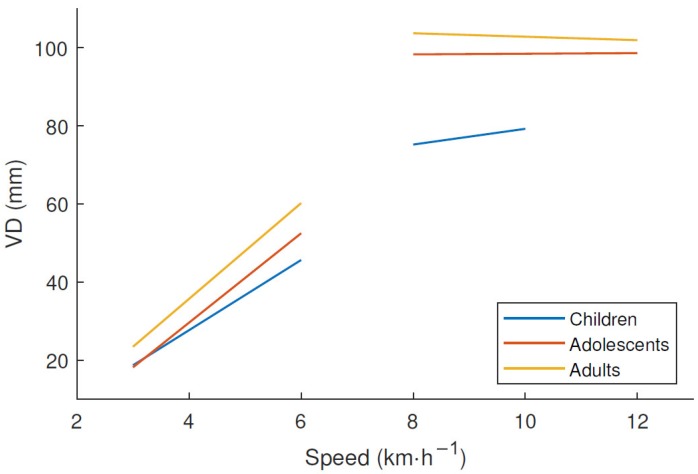
Vertical displacement of center of mass (VD) calculated from motion capture data of the marker at the lower back at different treadmill speeds with separate linear regressions representing walking and running with 10 subjects in each age group.

**Figure 4:**
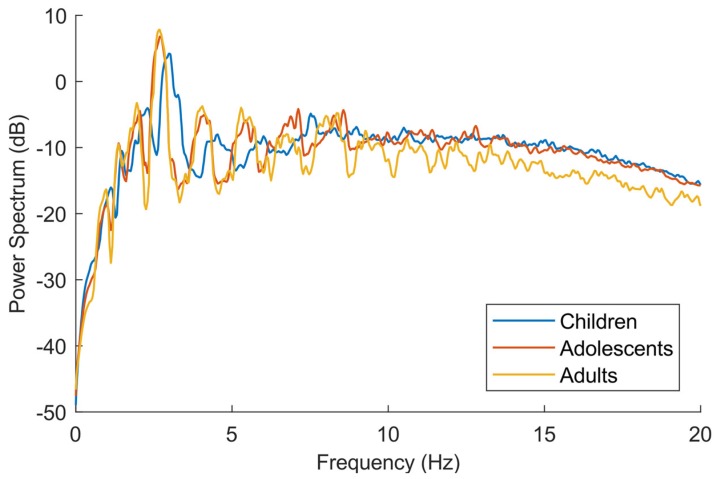
Power frequency spectrum of the acceleration from walking and running calculated from the reflective marker on the accelerometer using motion capture measurements with 10 subjects in each age group.

The characteristics of the subjects are shown in Table 1. It shows that the weight increased with age, but the increase in height between adolescents and adults was much smaller than that between children and adolescents. Figure 2 shows the estimated mass-specific internal, external, and total power for each age group. The children produced more internal but less external power during running compared to subjects from the two older groups, whereas there was no difference between the three age groups in total power. There was an increase in VD with increasing age at the same treadmill speed, as shown in Figure 3. After a linear increase in VD during walking and a major leap at the walking–running transition, there was no further increase in VD during running. The frequency spectrum seen in Figure 4 has clear peaks at 1–3 Hz, with children’s peaks at slightly higher frequencies than the other two groups.

**Figure 5:**
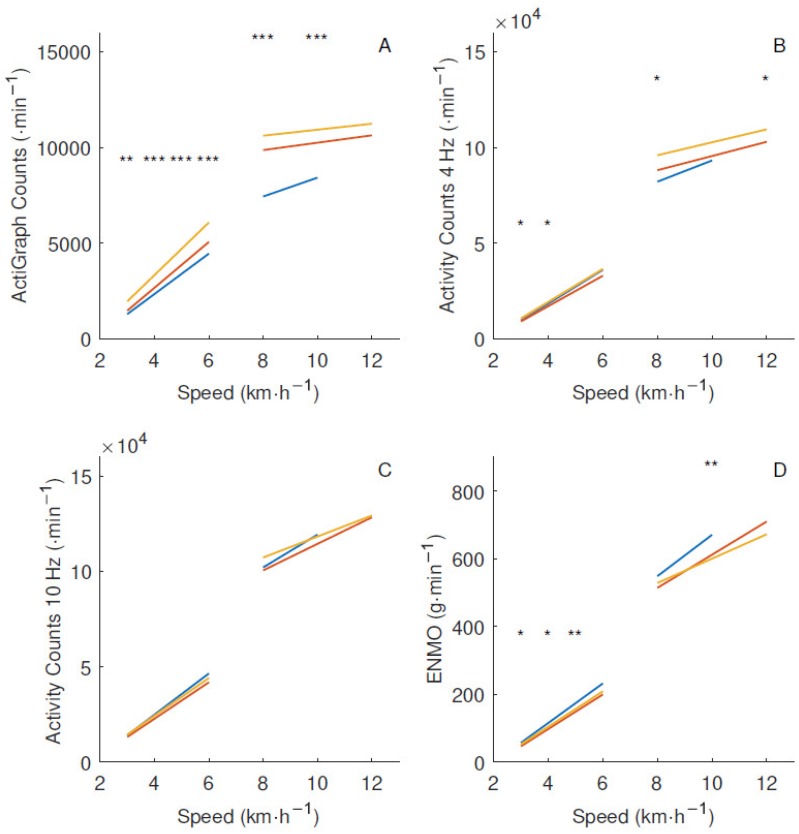
Number of counts at different treadmill speeds with separate linear regressions representing walking and running with 10 subjects in each age group. Children: blue, adolescents: red, adults: yellow. (**A**) ActiGraph filter; (**B**) 4 Hz filter; (**C**) 10 Hz filter; (**D**) Euclidian Norm Minus One (ENMO). Age-group difference from a one-way ANOVA at *: 0.01 ≤ *p* < 0.05, **: 0.001 ≤ *p* < 0.01, and ***: *p* < 0.001.

There was a linear increase in the number of counts generated with the AG filter during walking but only minimal changes across running speeds, as seen in Figure 5. With the wider filters, the counts kept increasing with running speed after a major increment with the walking–running transition. There were prominent statistical differences from the one-way ANOVA between the age groups across all treadmill speeds up to 10 km·h^−1^ with the AG filter, with *p* < 0.001 in a majority of the speeds. When the filter was widened to 4 Hz, the statistical differences decreased, and no differences with *p* < 0.05 remained between the age groups with the 10 Hz filter. In addition, the within-group CV decreased with wider filters among all age groups, as seen in Table 2. Compared to AC10, the statistical difference between the age groups was slightly higher with the unfiltered acceleration metric ENMO, where children produced more acceleration than adults. The shape of the relationship between speed and raw acceleration metric was similar to the 4 and 10 Hz filters.

The standardized coefficients from the multiple linear regression in Table 3 shows the relative contribution of VD and step frequency to the acceleration output. With a wider filter, the influence of the step frequency on the output increased from almost none to a similar level as the VD. The explained variation (R^2^) was consistently very high but decreased slightly when the filter was widened.

All subjects’ acceleration data output from each processing method and biomechanical variables are available as Supplementary Materials.

## 4. Discussion

The main result of the present study is that the narrow AG filter induced a measurement error by not accounting for variations in gait pattern across locomotion speeds and between individuals. Widening the frequency filter changed the relationship between locomotion speed and counts with improved assessment of high intensity. It also resulted in similar counts for different age groups. These findings were in line with what was expected from previous biomechanical research and confirmed our hypothesis. Implications of these results include more accurate measurements of physical activity and facilitated comparisons of physical activity levels between age groups, allowing age-independent calibration equations and cut-points for intensity levels. Today, there is a large number of calibration equations and intensity cut-points in children and adults with unknown comparability. The more accurate capturing of high intensity activities enables physical activity and its relation to different health aspects to be further studied.

The reduced individual variation and the removal of group difference in counts generated is explained by the progressively increasing part of step frequency, and therefore the internal work, being accounted for when allowing signals at higher frequencies to pass (Table 3). The original AG counts generated can be explained almost exclusively by the vertical displacement of the estimated center of mass (VD) during all locomotion speeds. Indeed, the narrow frequency filter seems to filter out all influence of the step frequency on the number of counts. Widening the filter to 4 Hz should capture most of the signal related to the heel strike and movement of the trunk as the step frequency during human locomotion rarely exceeds 4 Hz [13]. This signal should therefore capture most of the external work. Further widening of the frequency filter to 10 Hz seems to include other locomotion-related acceleration. The movement of the extremities in relation to the center of mass is more complex than the movement of the center of mass in relation to the environment. During the gait cycle, the extremities rotate and changes direction, which might affect the acceleration recorded at the hip with frequencies of a wider spectrum than just the step frequency. Therefore, the recorded acceleration with frequencies between 4 and 10 Hz may include a proportionally larger degree of internal work than the acceleration signal below 4 Hz.

The frequency power spectrum plot in Figure 4 also supports the assumption that the signal up to 4 Hz was primarily related to heel strike and that there was other locomotion-related acceleration between 4 and 10 Hz. The distinct peaks below 4 Hz represent the heel strike, and therefore the step frequency, at the different treadmill speeds. The line representing the children shifted approximately 0.5 Hz to the right compared to the other groups, indicating that their step frequency was generally 0.5 Hz higher. Adults and adolescents had approximately the same step frequency, which could be explained by the small difference in body height seen in Table 1. The echoing peaks visible at the frequencies between 4 and 10 Hz are to be considered as overtones of the step frequency. At about 9–12 Hz, the peaks were less distinguishable, which suggests that the part of the signal above ~10 Hz did not include more information relevant to the locomotion, a result that is in line with previous research [8,9]. A low-pass filtering of the accelerometer signal at 10 Hz also corresponds with the filtering level with the best agreement when comparing acceleration from accelerometers to reference methods such as motion capture systems and force plates [10,11].

Widening the filter slightly decreased the explained variation of the regression model in Table 3. The relationship between locomotion speed and internal power has more of a curvilinear shape compared to external power [14]. An accelerometry metric that is more influenced by step frequency might also show more of a curvilinear relation, which could lead to a lower explained variation of a linear regression. Furthermore, a processing method involving a wider filter could capture more noise, which would also explain the decrease in explained variability. Although this decrease was very small in our study, it may have had an impact on the results during free-living measurements due to more unwanted noise being captured from, for example, car riding or using power tools. In a free-living setting, factors other than the frequency filter might have a significant influence, such as a dead-band filter. Information in the signal above 10 Hz could be considered to be noise; however, in Figure 4, the amount of information in this range among children and adolescents were consistently higher than that among adults. This could explain why children produced more acceleration with the unfiltered ENMO.

Although AC10 might capture more noise, the variation between and within groups decreased compared to AG, as seen in Figure 5 and Table 2. This indicates that an important part of the large variation in AG counts for a particular activity is explained by measurement error, which is reduced by AC10 by more accurately capturing the acceleration signal within the entire range of frequencies related to locomotion. The preferred step frequency is influenced by body height; being taller is associated with longer strides and lower step frequency at a particular locomotion speed [21]. Therefore, a wider frequency filter would not only decrease the age-group differences but also the within-group variation related to different body height by more accurately capturing physical activity with varying step frequency.

Differences in internal/external work between children and adults described by Schepens et al. seems to diminish after the age of 10 [12]. In our results, although there were no differences in step frequency between adolescents and adults, adolescents yielded less counts with all the investigated filtering levels compared to adults. This could be explained by adolescents running and walking with the same stride length but less VD (Figure 3). This means that the trajectory of the estimated center of mass is flatter among adolescents, and that they do not perform as much vertical work as adults although the stride length is the same. The majority of the acceleration related to locomotion is vertical, and a decrement of this would lead to lower amount of counts [4].

The absolute values of acceleration from AG and ENMO as well as the difference between age groups with these methods are consistent with previous findings [7,22,23]. The differences between the output from AG and ENMO is to a large extent caused by the frequency filter, though other components of the processing than those that have been discussed previously could have an impact. After filtering, down-sampling, and truncation, AG counts are generated by aggregating the absolute acceleration over a specific time period, referred to as the epoch length, followed by the calculation of vector magnitude according to the Euclidean norm [3]. The ENMO processing, on the other hand, involves zeroing all negative acceleration after subtracting 1 g from the vector magnitude [18]. This removes all negative acceleration collected between 0 and −2 g. Children rely more on their step frequency than their amplitude of acceleration to produce the accelerometer output compared to adults. Removing a constant amount of acceleration amplitude could make the step frequency relatively more influential compared to the amplitude (as seen in Table 3), which could explain the larger amount of acceleration measured among children with ENMO.

The shape of the distribution of AG counts in Figure 5A is similar to the external power and VD in Figure 2 and Figure 3, respectively, which has previously been described as the plateau phenomenon [4]. AG counts represent the external power and VD quite accurately, but the relationship between these variables and speed makes it inappropriate to use as a measurement of vigorous physical activity. With the plateau, it is impossible to distinguish between different running speeds. The counts from the wider filters continued to increase with higher speeds (Figure 5B,C), with a relationship similar to the total power in Figure 2. A wider filter would enable physical activity to be distinguished at high intensities in a way that has not been possible before due to the plateau phenomenon. Vigorous physical activity, even in small doses such as high intensity interval training, has been shown to improve health [24]. An acceleration metric that is more sensible to vigorous physical activities might facilitate the studying of these effects further.

This study compared data from accelerometers placed on the hip only. Theoretically, sensors placed more distal to the center of mass could measure more signals related to the internal work relative to the external. Therefore, other levels of data filtering could be more appropriate to capture optimal acceleration at different sensor placements from a biomechanical perspective. Only two data filters were used in addition to the standard AG filter as well as unfiltered data to compare the outcome between the age groups. An optimal filtering frequency should accurately capture vigorous physical activity, enable age-independent calibration, and minimize recorded noise. In addition, walking and running on a treadmill were the only activities investigated. The counts captured during treadmill locomotion have been shown to differ compared to over ground [25]. Furthermore, even larger age-group differences have been shown with other activities compared to locomotion [7].

The methodology used for capturing mechanical work herein were a modification of a method designed for running. Therefore, this might not accurately represent the amount of work related to walking, which is usually described as an inverted pendulum instead of the spring mass model used to describe running [20]. During walking, there is a component of internal work related to the double support part of the gait cycle, which was not accounted for in the current model [12]. In addition, instead of using the real center of mass, a fixed marker on the lower back was used as an estimation. Acceleration measured at the lower back during locomotion is considerably lower than acceleration closer to the ground [26]. Even though the actual numbers of the internal and external power might not be accurate, the relation between the age groups is similar to previous research [12,14].

A total of 28 one-way ANOVAs were performed on the material. Using a Bonferroni adjustment to decrease the risk of making a type I error, the usual 0.05 significance level would be set to 0.0018. In that case, only the highest significances at *p* < 0.001, indicated by *** in Figure 5, would be considered real differences. Nevertheless, the lower level significances clearly show the pattern of decreasing differences with a wider filter. Bonferroni adjustments have been critiqued for being unnecessary and creating more problems with statistical interpretation [27]. A Bonferroni adjustment would dramatically increase the risk of type II errors, which is just as relevant as type I errors in the current study.

AG counts have been the dominating method for objective measurement of physical activity for 20 years and many researchers base their understanding of physical activity on this metric. The modified method could have expressed the aggregated acceleration in m/s or mean acceleration of the epochs in mg’s similar to other methods [18]. Instead, the conversion equivalent to 2.13 g to 8-bit resolution represented by counts was kept to facilitate comprehension and future implementation in clinical research.

The added value of the present findings is the effect of a frequency filter on differences in accelerometer output between age groups in a controlled lab environment and in relation to biomechanical variables. The study design enables both investigating the effect of a frequency filter on accelerometer outputs and explaining why the output would differ. The results from this study promote a re-evaluation of the numerous calibration equations that have been previously used. Several methods have been proposed to improve the interpretation of AG counts, including nonlinear regression equations, two-regression models, regressions with age as a factor, or machine learning algorithms [1,28]. Instead of developing ways of making AG counts more interpretable, the current study targets the process generating the counts to achieve a more accurate representation of physical activity from a biomechanical perspective.

Future studies should investigate age differences in accelerometer data from other sensor placements than the hip as well as using a wide range of different frequency filters in order to find one that is optimal for free-living measurements of physical activity. Possible negative effects of wider frequency filters in a free-living setting with regard to more noise being captured is also an important topic for future studies. The relationship between energy expenditure and acceleration outputs with different filters should be investigated as should activities other than treadmill locomotion.

## 5. Conclusions

Widening the frequency filter involved in the processing of acceleration with ActiGraph improves assessment of physical activity intensity, reduces individual variation, and removes age-group differences in the number of counts generated during locomotion. This is explained biomechanically by more accurately capturing individual gait patterns across locomotion speeds. These findings confirm our hypothesis and suggests that age-independent calibration equations are possible with an optimal processing of acceleration data.

## Figures and Tables

**Table 1:** Subjects’ characteristics.

	Children	Adolescents	Adults
N (% female)	10 (30%)	10 (50%)	10 (50 %)
Age (years)	10.1 (0.6)	15.1 (0.4)	29.2 (6.6)
Weight (kg)	32.0 (6.3)	59.5 (6.3)	68.4 (10.4)
Height (cm)	140 (7.5)	170 (8.3)	174 (10.1)
Upper arm (cm)	24.6 (1.4)	29.2 (2.0)	30.5 (1.8)
Forearm (cm)	20.3 (1.4)	24.9 (1.4)	26.1 (2.0)
Thigh (cm)	34.2 (4.7)	43.0 (3.2)	44.6 (1.6)
Shank (cm)	34.0 (2.5)	41.8 (3.3)	42.0 (2.6)

Group mean (standard deviation).

**Table 2:** Within age-group mean coefficients of variation across all treadmill speeds for each processing method.

Method	Children	Adolescents	Adults
AG	18.3%	17.4%	13.0%
AC4	11.0%	12.6%	9.3%
AC10	10.8%	12.0%	8.7%
ENMO	10.5%	12.1%	11.1%

AG: ActiGraph counts, AC4: 4 Hz Activity counts, 10 Hz: Activity counts, ENMO: Euclidian norm minus one.

**Table 3:** Standardized coefficients and total explained variation (R^2^) from linear regressions between the output from the different data processing methods as dependent variables and vertical displacement of center of mass (VD) and step frequency as independent variables.

Method	Standardized Coefficients		
	VD	Step Frequency	R^2^
AG	0.937	0.064	0.979
AC4	0.656	0.378	0.970
AC10	0.535	0.492	0.950
ENMO	0.471	0.535	0.911

AG: ActiGraph counts, AC4: 4 Hz Activity counts, 10 Hz: Activity counts, ENMO: Euclidian norm minus one.

## Supplementary Materials

All subjects’ acceleration data output from each processing method and biomechanical variables are available online at http://www.mdpi.com/1424-8220/18/10/3399/s1.
